# Compression and bending performance of novel 3D printed mixed-star negative Poisson’s ratio metamaterials using CCD-based optimization and finite element analysis

**DOI:** 10.1038/s41598-026-47212-3

**Published:** 2026-04-16

**Authors:** Sohail Gohar, Babar Ashfaq, Hamza Qayyum, Malik Hassan, Ghulam Hussain, Mohammed Alkahtani

**Affiliations:** 1 Mechanical Engineering Department, Sarhad University of Science and Information Technology, Peshawar, 25000 Pakistan; 2 Faculty of Mechanical Engineering, GIK Institute of Engineering Sciences & Technology, Topi, 23460 Pakistan; 3 School of Engineering, University of Guelph, Thornbrough Building, Guelph, ON N1G 2W1 Canada; 4 Mechanical Engineering Department, Faculty of Engineering, University of Bahrain, Sakhir, 32038 Kingdom of Bahrain; 5 Department of Industrial Engineering, College of Engineering, King Saud University, P.O.Box 800, Riyadh, 11421 Saudi Arabia

**Keywords:** Negative Poisson’s ratio, Mixed star cell, Cell optimization, Compression, Flexural, Mechanical Properties, Engineering, Materials science, Mathematics and computing

## Abstract

Auxetic structures, recognized for their lightweight design, auxeticity, and exceptional mechanical properties, have garnered considerable attention from the engineering sector. The auxeticity and associated mechanical traits of these structures depend on the negative Poisson’s ratio (NPR) principle. These structures comprise a collection of repetitive unit cells, the size of which is crucial for achieving the desired performance. This study introduces a high-performing auxetic structure based on a unit cell named Mixed Star and optimizes its performance using a hybrid statistical-numerical approach. Considering the length, height, thickness, and inclination of the wall as cell parameters, a detailed test plan was generated through a statistical approach. A series of mixed-star Metamaterial (MSM) structures were modeled according to the Design of Experiment plan, and their mechanical performance, specifically energy absorption, modulus, and strength in compression and bending, was evaluated using a validated FEM model. The collected results were analyzed, revealing key effects along with their nature. In-depth analysis of the findings indicated that the auxetic behavior of the structure is closely associated with the size of its unit cell, highlighting the need for an optimized cell size to enhance structural performance. By applying an appropriate approach, the optimum cell size was determined while considering both compression and flexural loads, resulting in substantial gains in auxeticity and mechanical performance, depending on the response parameter and loading condition. This study emphasizes the potential of a hybrid statistical-numerical approach in optimizing the geometry of NPR structures to achieve superior performance, providing a valuable framework and paving the way forward for future research.

## Introduction

Additive Manufacturing (AM) has emerged as a transformative technology enabling the fabrication of complex cellular structures with high design flexibility, minimal material waste, and tailored properties^1,2^. These outstanding properties make them suitable for various applications, including aerospace^3,4^, transportation^5^, architecture^6^, and biomedical fields^7^. Recently, a new type of mechanical structures known as auxetic structures has been developed^8^. These structures have garnered significant interest from both academia and industry due to their unique mechanical properties^9^. The key to their exceptional performance lies in their Negative Poisson’s Ratio (NPR). The NPR materials expand laterally when stretched, while the materials with a positive Poisson’s ratio contract under the same conditions^10^.

Auxetic structures have demonstrated superior mechanical performance compared to traditional honeycomb structures, including enhanced bending strength and improved stress distribution^11,12^ as well as better shear properties^13^, indentation resistance^14^, impact behavior^15^, energy absorption^16,17^, vibration suppression^18,19^ and compressive strength^20–22^. These characteristics make NPR structures highly suitable as sandwich cores in civil and military applications such as aircraft, automobiles, and satellites, with improved damping and acoustic performance^23,24^.

Auxetic Structures, as described by Gibson and Ashby^25^, are porous formations that enable efficient material distribution to enhance mechanical performance also reported by Banhart et. al.,^26^. These structures are known for their exceptional mechanical performance, and it is strongly influenced by the size, shape, and topology of unit cells^27–29^ which has led to the development of various design and optimization approaches to further improve their mechanical behavior. In recent years, structural optimization has emerged as an effective approach for designing advanced materials and structures^17,30^. It has been widely applied to auxetic structures to achieve objectives such as maximizing bulk modulus^31,32^, optimizing thermal expansion^33,34^, achieving high NPR^35^, enhanced multifunctional properties^36,37^ and excellent functionally graded performance^38,39^.

Researchers have employed various methods to enhance the in-plane stiffness of NPR structures. Nian et al.^40^ demonstrated that graded cellular structures significantly improve energy absorption under bending with specific energy absorption increase up to 48.9%. Lu et al.^41^ added a vertical rib to the re-entrant structure, enhancing stiffness while Fu et al.^42^ combined rhombic and re-entrant structures to further improve mechanical response. Chen et al.^43^ extended 2D re-entrant structures into 3D configurations, and Dong et al.^44^ reported improved NPR behavior through the addition of sinusoidal or vertical ribs. Han et al.^45,46^ further highlighted that geometric parameters, particularly thickness and height, play a critical role in determining structural performance. Moreover, advanced auxetic designs such as gradient-variable Poisson’s ratio structures and hierarchical metamaterials have demonstrated significant improvements in energy absorption and buckling resistance^47,48^. Parametric and statistical studies have further emphasized the importance of geometric optimization as Qi et al.^49^ showed that Design of Experiments (DoE) and meta-modeling approaches can efficiently explore the design space and identify optimal configurations for improved structural performance.

In another study^50^, a mixed star structure (MSM) was proposed, demonstrating superior mechanical performance compared to existing auxetic designs. Though the importance of cell optimization has been highlighted in the literature, the reported results are rather limited and valid only for specific structures. While the optimum cell parameters may vary with the cell geometry and type of loading. Moreover, the past studies aiming on cell geometry, which were very few, did not consider the interactive (or combined) effects of cell parameters. Therefore, further investigations become imperative to address these points to widen the knowledge base for a broader range of engineering applications. To this end, the authors executed investigations by adopting a hybrid statistical-numerical approach. This approach is systematic and more economical in contrast to the approaches adopted in previous studies. This approach relies on a validated FEA model to evaluate the mechanical performance of structures.

Highlighting the importance of statistical-numerical approaches for reliable results opens possibilities to investigating complex interactions between multiple input variables on key output variables with fewer experiments. In this research, a comprehensive plan composed of 31 runs with varied cell parameters was developed using the CCD statistical method. The mechanical performance of these structures was evaluated using the validated model of FEA. The key cell parameters significantly influencing mechanical performance and the nature of their effect were identified. Numerical optimization was performed on the results to realize optimum cell geometry, which demonstrated substantially higher performance than the benchmark structure. These findings offer valuable insights, providing a basis for designing and developing advanced auxetic structures with improved performance for various engineering applications.

## Materials and methods

**Fig. 1:**
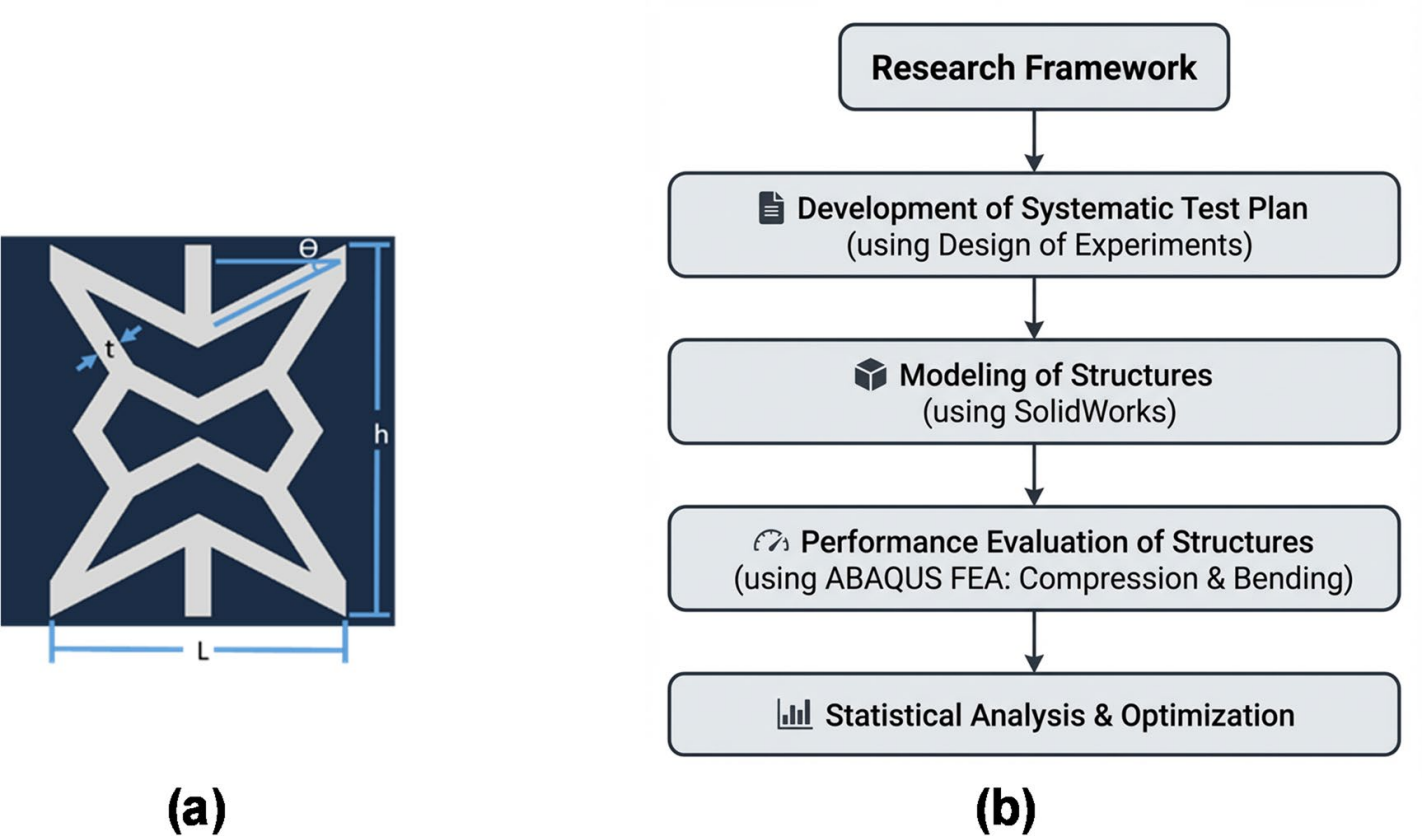
(**a**) Geometry parameters of Unit Cell of Auxetic Mixed Star Structure (**b**) Framework of Study.

Figure 1a shows the unit cell of the mixed star metamaterial (MSM) adopted as a test geometry in the present study. The relative density of the MSM can be expressed in terms of its geometrical parameters using Eq. (1).

1 $$\text{Relative Density }=\frac{{p}^{*}}{{p}_{s}}=\frac{2\mathrm{t}\left({l}_{1}+{l}_{2}+{l}_{3}+{l}_{4}\right)}{2{l}_{2}^{2}\mathrm{cos}{\theta }_{1}\mathrm{sin}{\theta }_{1}+4{l}_{4}^{2}\mathrm{cos}{\theta }_{2}\mathrm{sin}{\theta }_{2}+2{l}_{3}^{2}\mathrm{cos}{\theta }_{3}\mathrm{sin}{\theta }_{3}+2{l}_{4}\mathrm{cos}{\theta }_{2}\left[{h}_{1}-2{l}_{4}\mathrm{sin}{\theta }_{2}\right]}$$

As illustrated in Fig. 1b, the study was conducted in four phases: (i) Development of a systematic test plan, (ii) Modelling of structures according to the test plan, (iii) Performance evaluation of structures using FEA, and (iv) Analysis and optimization. The Design of Experiments (DoE) approach formulated a test plan. Structures for each trial were modeled using SolidWorks software. Mechanical performance of structures was assessed under compression and bending loads via Finite Element Analysis using ABAQUS CAE. The performance parameters, namely energy absorption, modulus, and strength, were derived from numerical simulations and analyzed statistically to quantify the performance of each design. Minitab 2022 was used to perform statistical analysis. The framework of this research study is illustrated in Fig. 1b.

### Design of experiments (DoE)

The experiment set was designed utilizing a Central Composite Design (CCD) approach, a type of Response Surface Methodology (RSM). It is considered one of the most robust techniques in the DOE approach to explore the interaction between independent variables, model the system mathematically, and minimize the number of experimental runs^51^. CCD was implemented using four geometric parameters of MSM: i.e., cell wall thickness, unit cell length, unit cell height, and angle of the inclined wall as shown in Fig. 1. These parameters were specifically chosen based on the methodology applied to star structures in a previous study^48^. Each parameter was varied over 3 levels as illustrated in Table 1. Whereas the generated experimental runs for compression and flexural testing are given in Table 2.

**Table 1:** Levels of geometrical parameters.

Parameter	Symbol	Unit	Low-level	Mid-level	High-level
Length of a unit cell	L	mm	9	12	15
Wall thickness	t	mm	1	2	3
Angle of inclined wall	Ɵ	degree	10	25	40
Height of unit cell	h	mm	9	12	15

**Table 2:** Test plan for compression and flexural testing.

Std order	Run order	L(mm)	t (mm)	Theta (deg)	h (mm)
26	1	12	2	25	12
3	2	9	3	10	9
6	3	15	1	40	9
4	4	15	3	10	9
27	5	12	2	25	12
13	6	9	1	40	15
25	7	12	2	25	12
10	8	15	1	10	15
2	9	15	1	10	9
5	10	9	1	40	9
22	11	12	2	40	12
24	12	12	2	25	15
21	13	12	2	10	12
31	14	12	2	25	12
1	15	9	1	10	9
20	16	12	3	25	12
11	17	9	3	10	15
7	18	9	3	40	9
29	19	12	2	25	12
17	20	9	2	25	12
19	21	12	1	25	12
8	22	15	3	40	9
9	23	9	1	10	15
18	24	15	2	25	12
14	25	15	1	40	15
28	26	12	2	25	12
15	27	9	3	40	15
12	28	15	3	10	15
30	29	12	2	25	12
23	30	12	2	25	9
16	31	15	3	40	15

### CAD modelling and FEA procedure

**Fig. 2:**
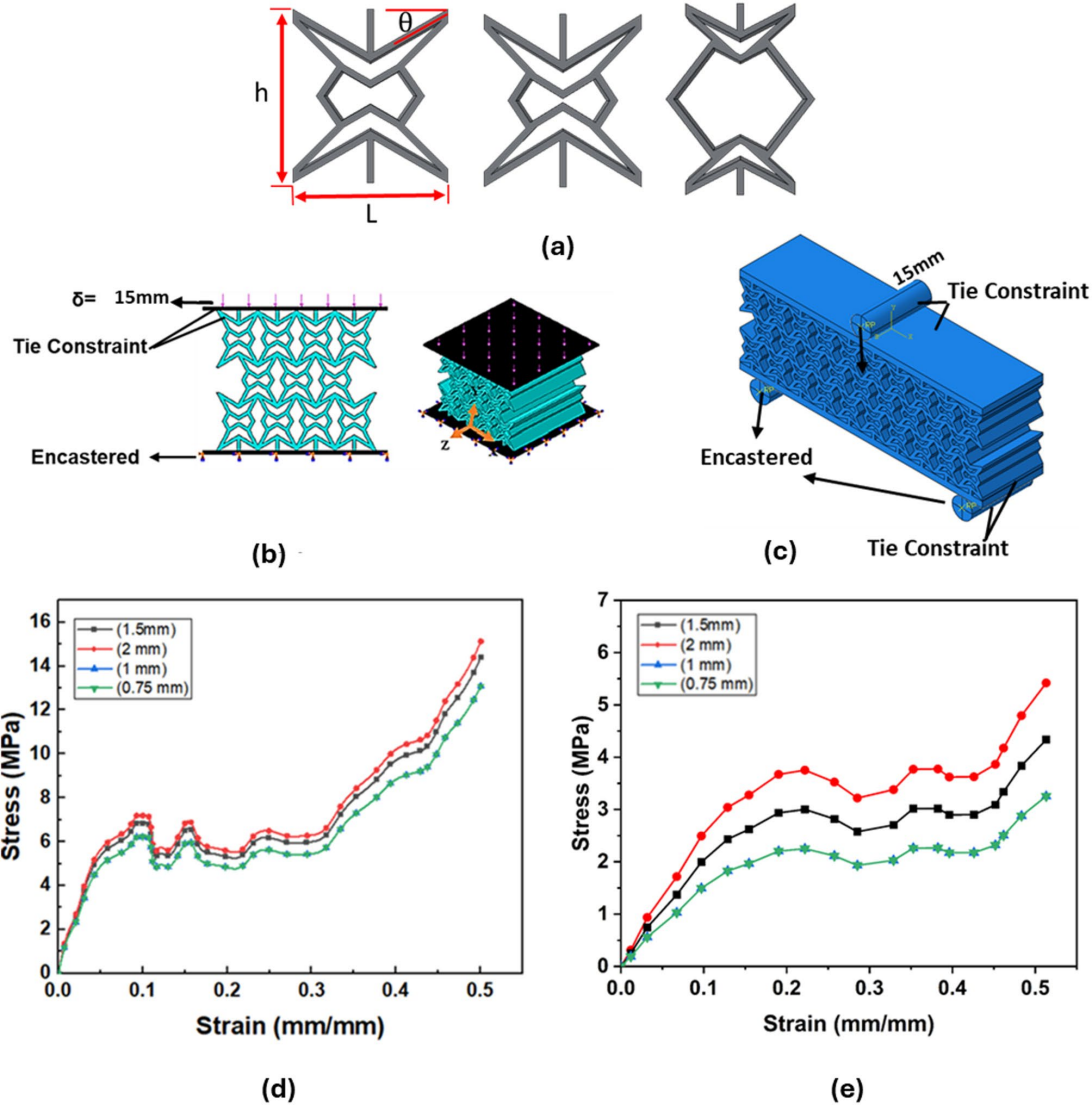
(**a**) Representatives designs from DOE (**b**) FEA model for compression (**c**) FEA model for bending (**d**) mesh sensitivity analysis for compression (**e**) mesh sensitivity analysis for bending.

Following the generation of the experimental plan, the next step involved CAD modelling of structures for the respective experimental runs. A total of 31 models were created, with representative models shown in Fig. 2a. The mechanical performance of the MSM was subsequently analyzed using Finite Element Analysis (FEA) with the ABAQUS Explicit model. A detailed representation of the FEA procedure for compression and flexural testing is shown in Fig. 2b-c.

Firstly, the 3D models were created in SolidWorks and imported into ABAQUS CAE, where a mesh sensitivity analysis was conducted to find the optimal mesh size that minimizes computation time and effort without sacrificing numerical accuracy. The initial results indicated that a 1 mm mesh size was appropriate, as shown in Fig. 2d-e. For compression testing, the MSM dimensions were kept at 40 × 40 × 45 mm, according to the ASTM D-1621 standard. The structure comprised two rigid face sheets firmly attached to the top and bottom of an MSM auxetic core using tie constraints. The bottom sheet was fixed in all degrees of freedom with the ENCASTRE boundary condition, while the movement of the top face sheet was restricted in all directions except Y, with a 15 mm incremental displacement applied in the negative Y direction. A coefficient of friction of 0.3 was used between the top plate and the auxetic core. Additionally, self-contact was enabled to account for surface interactions within the auxetic cores. This modeling approach yielded consistent and accurate results, aligning with experimental data as shown in previous research^50^. The MSM dimensions for compression testing remained 40 × 40 × 45 mm, as per ASTM D-1621.

Similarly, for the flexural testing of the MSS structures, samples were designed according to the guidelines of the ASTM-D790 standard with dimensions of 110 × 15 × 45 mm. A mesh size of 1 mm was also found to be optimal for the flexural tests. A 15 mm displacement in the y-direction was applied to the top roller while all other degrees of freedom were fixed. An ENCASTRE boundary condition was applied to the two bottom side rollers, which formed a span of 72 mm. General contact interactions were used here with no slipping to simulate the interaction between the rollers and the auxetic structure. A reference point was used to extract the force–displacement response of each structure. The compression and flexural material properties of ABS were experimentally evaluated as given in Table 3 and assigned to the MSM core in respective testing numerical frameworks. A C3D10 ten-node quadratic tetrahedral element type was chosen because of its versatility and ability to fill complex geometries without sacrificing result quality^50,52^. It is pertinent to note that the overall size of the specimen geometries was maintained, hence the number of cells were adjusted to address this.

**Table 3:** Material properties of ABS.

Material	Flexural modulus (MPa)	Compressive modulus (MPa)	Flexural yield stress (MPa)	Compressive yield stress (MPa)	Poisson’s ratio	Density (kg/m^3^)
ABS	2100	1600	55	52	0.35	1050

### Predictive models

Response Surface Methodology (RSM) provides a means to model and analyze the effects of various variables on a response factor. Herein, Analysis of Variance (ANOVA) and RSM were used to identify significant geometrical parameters and formulate predictive 2nd order predictive models using four factors for performance prediction of MSM, respectively. A 2nd order general expression obtained from ANOVA is given here in Eq. 2^53^.

2 $$\begin{aligned} Y = & b_{0} + b_{1} x_{1} + b_{2} x_{2} + b_{3} x_{3} + b_{4} x_{4} + b_{{12}} x_{1} x_{2} + b_{{13}} x_{1} x_{3} \\ & + b_{{14}} x_{1} x_{4} + b_{{23}} x_{2} x_{3} + b_{{24}} x_{2} x_{4} + b_{{34}} x_{3} x_{4} + b_{{11}} x_{1}^{2} + b_{{22}} x_{2}^{2} \\ & + b_{{33}} x_{3}^{2} + b_{{44}} x_{4}^{2} \\ \end{aligned}$$

### Cell optimization

The multi-objective optimization was carried out using the desirability-based approach, in which each response (energy absorption, modulus, and strength) was independently transformed into a desirability function ranging from 0 to 1 using a “larger-is-better” criterion. Equal importance was assigned to all responses to ensure balanced optimization without bias toward a specific mechanical property. The overall desirability was computed as the geometric mean of the individual desirability, and the cell geometry corresponding to the maximum composite desirability was identified as the optimal solution.

## Results and discussions

### Compression behavior and properties

**Fig. 3:**
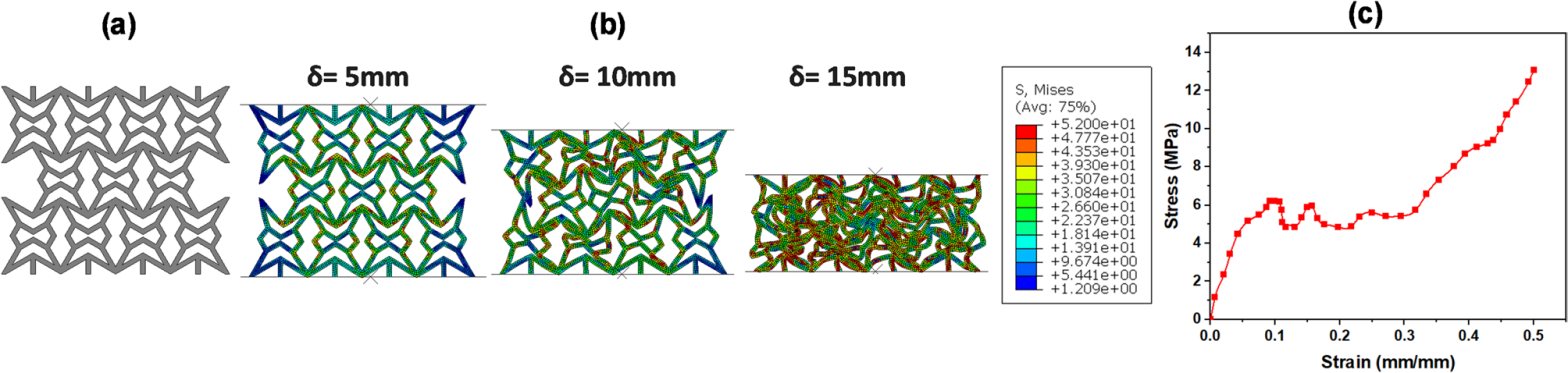
FEA results of MSM (**a**) Structure geometry (**b**) Deformation modes (**c**) A representative stress–strain curve.

FEA simulations were conducted to evaluate the compression performance of all 31 MSM cell designs listed in Table 2, a representative of which is shown in Fig. 3a-b. The FEA results revealed that the MSM exhibited three prominent peaks in the stress–strain curve between 0.1 and 0.2 mm/mm strain in Fig. 3c. These variations are likely due to wall buckling rather than the collapse of individual rows. It was observed that the collapse initiated from the top row and propagated towards the moving end, ultimately leading to the collapse of the fixed end. The stress increased monotonically with strain from 0 to 0.5 mm/mm, demonstrating stable mechanical performance under the applied compression load.

The compressive response of MSM was evaluated in terms of compression modulus and compression strength as listed in Table 4. Moreover, the specimens were compressed till the densification strain so that the EAC of the structures could be estimated. According to^54^, the densification strain is the strain where the slope of the tangent to the stress–strain curve becomes equal to the slope of the elastic region of the curve. As reported in^55^, it is defined as the last local minimum before which the stress rises steeply. Using both the above-mentioned methodologies, the densification strain of MSM was calculated to be in a range between 0.23 and 0.4. The EACs were calculated using Eq. 3.

3 $$W= {\int }_{0}^{\varepsilon }\sigma (\varepsilon )d\varepsilon$$

where, *W* = Energy absorption capacity; $\sigma \left( \varepsilon \right)$= Stress–Strain Curve

**Table 4:** Compression properties from 31 tests.

Test #	L (mm)	t (mm)	θ^o^	*h*(mm)	Compressive properties		
					Energy absorbed (kJ/m^3^)	Compression modulus (MPa)	Compression strength (MPa)
1	12	2	25	12	2.750	15.50	2.10
2	9	3	10	9	5.085	41.70	3.40
3	15	1	40	9	2.800	15.00	2.20
4	15	3	10	9	20.600	145.00	7.20
5	15	2	25	15	22.550	165.00	8.10
6	9	1	40	15	5.015	41.20	3.15
7	12	2	25	12	2.750	15.50	2.10
8	15	1	10	15	22.650	165.00	8.20
9	15	1	10	9	2.020	8.20	0.30
10	9	1	40	9	10.000	135.00	6.10
11	12	2	40	12	2.500	35.00	9.60
12	12	2	25	15	5.150	46.50	30.50
13	12	2	10	12	2.015	5.55	8.10
14	12	2	25	12	2.750	15.50	2.10
15	9	1	10	9	2.055	8.40	8.25
16	12	3	25	12	5.050	42.50	3.20
17	9	3	10	15	3.300	30.00	1.30
18	9	3	40	9	6.350	64.00	3.30
19	12	2	25	12	2.750	15.50	2.10
20	9	2	25	12	17.300	115.00	3.50
21	12	1	25	12	2.620	29.00	1.70
22	15	3	40	9	16.500	130.00	6.80
23	9	1	10	15	13.600	115.00	5.20
24	15	2	25	12	16.400	135.00	6.20
25	15	1	40	15	13.800	120.00	4.50
26	12	2	25	12	2.750	15.50	2.10
27	9	3	40	15	3.200	65.00	1.50
28	15	3	10	15	14.40	135.00	6.30
29	12	2	25	12	2.750	15.50	2.10
30	12	2	25	9	16.70	145.00	5.20
31	15	3	40	15	4.10	100.00	3.80

#### Key influential cell parameters and their response surface plots

ANOVA is a statistical tool that is used to identify the influential parameters. This analysis was performed to sort out the significance of cell parameters namely *l, t, h,* and *θ* of MSM. ANOVA results for all the responses are outlined in Table 5. *p*-value serves as an indicator of the relative significance of a factor, defining the threshold for statistical significance. A *p*-value exceeding 0.05 suggests that the associated factor is statistically insignificant, while a lower *p*-value indicates a stronger influence on the response. Similarly, F-value (Fischer Test Values) provides insights into the effects of factors, with larger F-values signifying a greater impact on the response.

**Table 5:** Analysis of variance for energy absorbed, modulus and strength under compression.

EA	DF	Adj SS	Adj MS	F-Value	*p*-Value
L	1	0.0001	0.0001	1.23	0.041
h	1	2.2025	2.2015	1.74	0.035
t	1	4.133	4.1328	3.27	0.011
Theta	1	1.960	1.9602	1.5	0.031
h^2^	1	5.87	5.8780	4.6	0.047
L*t	1	8.50	8.5045	6.72	0.020
L*Theta	1	15.200	15.2005	6.72	0.020
t*h	1	5.204	5.2041	4.11	0.049
Error	16	25.319	1.5824		
Lack-of-Fit	10	25.319	2.5319	0.74	0.69
Pure Error	6	11.50	1.92		

CM	DF	Adj SS	Adj MS	F-Value	*p*-Value
L	1	8359.2	8359.2	1.25	0.036
t	1	1538.3	1538.3	6.75	0.014
Theta	1	0.1	0.1	0.56	0.048
h	1	1018.5	1018.5	0.64	0.043
t*t	1	1020.2	1020.2	7.35	0.001
L*t	1	7686.9	7686.9	4.80	0.044
h*Theta	1	4539.4	4539.4	2.84	0.027
l*h	1	3297.6	3297.6	2.06	0.011
Error	16	4400.1	275.1		
Lack-of-Fit	10	2400.5	639.35	0.72	0.71
Pure Error	6	2000	333.3		

CS	DF	Adj SS	Adj MS	F-Value	*p*-Value
L	1	5.34	5.336	1.40	0.043
t	1	366.0	366.302	8.65	0.019
h	1	65.36	65.361	1.54	0.023
h*h	1	240.22	240.220	5.67	0.030
l*l	1	554.78	554.781	13.10	0.002
t*t	1	536.56	536.558	5.32	0.030
L*Theta	1	447.32	447.323	10.56	0.005
t*h	1	317.73	317.731	7.50	0.015
Error	16	677.65	42.353		
Lack-of-Fit	10	115	11.50	0.63	0.76
Pure Error	6	95.0	15.83		

For EA all selected geometric parameters were statistically significant, including the binary and square interactions between them. Among these factors, thickness emerged as the most influential parameter, as it appeared in all interactions and exhibited a *p*-value ≤ 0.05. For CM, all individual parameters were also significant, with *p*-values ≤ 0.05. Thickness again demonstrated notable significance, particularly in binary and square interactions, with a *p*-value of 0.001. Lastly, for CS, the parameters *l*, *t,* and *h* were the most significant factors, with *p*-values of 0.043, 0.019, and 0.023, respectively.

**Fig. 4.**
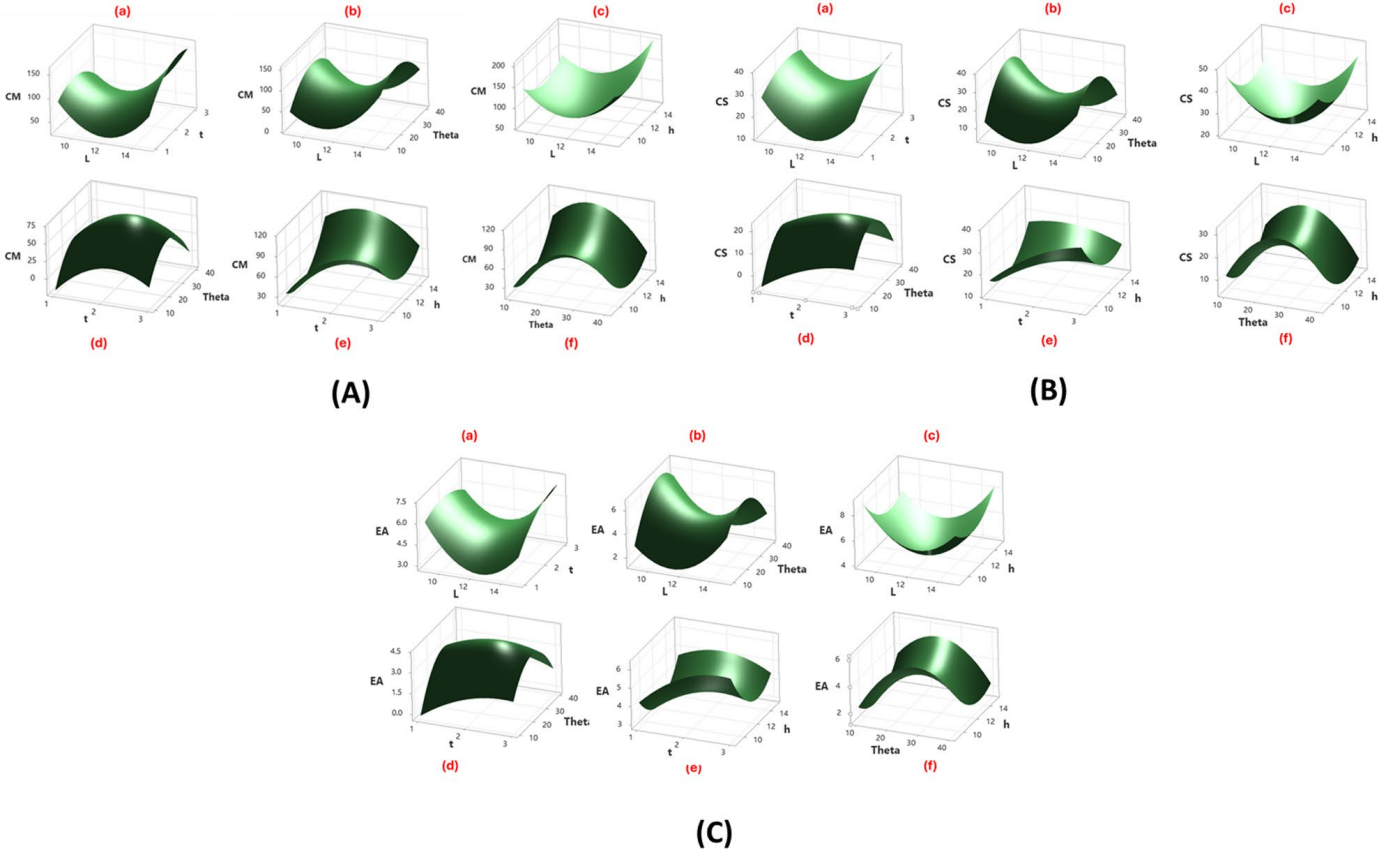
3D surface plots for in compression (**A**) Compressive modulus (**B**) Compressive strength (**C**) Energy absorption.

Figure 4A–C presents 3D surface plots illustrating the influence of geometric parameters on CM, CS, and EA under compression. CM increases with both thickness and length, with length exhibiting a stronger influence, while non-linear dependencies are observed in thickness-angle and angle-height interactions. CS peaks at specific angle-length combinations, and EA improves primarily with increasing thickness and height. Additional surface plots displaying similar trends are provided in the supplementary material.

The thickness-height interaction is particularly noteworthy, as increased wall thickness enhances local flexural rigidity of cell ligaments, delaying buckling, while greater cell height facilitates stress redistribution across multiple layers. When acting concurrently, these parameters promote a stable progressive collapse mode, resulting in improved stiffness, strength, and energy absorption under both loading conditions.

### Flexural behavior and properties

**Fig. 5:**
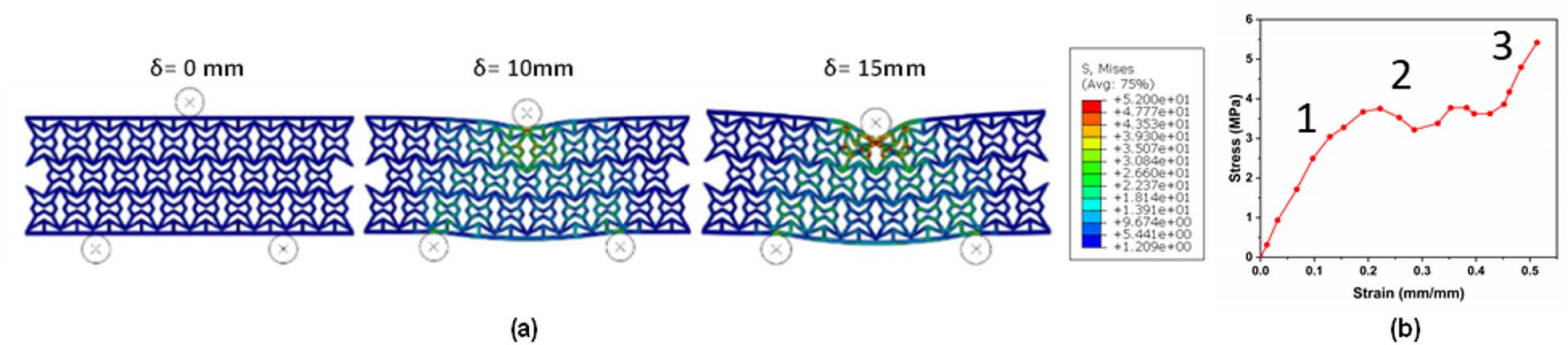
Flexural analysis using FEM (**a**) Deformation modes (**b**) Stress–strain curve.

FEA simulations of 3-point bending tests were performed for all cell geometries; one such representative simulation is illustrated in Fig. 5, and the recorded response is shown in Fig. 5b. It is found that the MSM unit cells in the close vicinity of the indenting roller undergo local deformations, whereas the cells far from the roller are subjected to minimal deformation. It was also observed that the cell close to the roller tended to rotate toward the center of the structure due to the compressive component of the bending load, whereas the cells near the supports were in tension. This rotation of the cells towards the center further highlights the auxetic nature of the designed geometry. The flexural response of MSM structures can be divided into three phases, the first phase can be characterized as the elastic buckling phase, where the ligaments at the central region try to take up the entire load while gradually transferring the load to neighboring ligaments. In the second phase, the structure gets deformed and the continuous humps in the stress–strain curve were observed. The gradual humps in the curve were observable in this region and may be attributed to the plastic buckling of the hinges. As the displacement is further increased, the self-contact of the ligaments causes the local densification of the unit cells at the center, which causes a sharp increase in the load-bearing capacity of the structure at higher displacements (region 3). The flexural properties of the MSM evaluated through the FEA framework are listed in Table 6.

**Table 6:** Flexural properties of MSM.

Test #	L (mm)	t (mm)	θ^o^	h(mm)	Flexural properties		
					Energy absorbed (kJ/m^3^)	Flexural modulus (MPa)	Flexural strength (MPa)
1	12	2	10	12	0.980	16.50	6.00
2	12	2	25	12	0.150	3.00	0.90
3	9	1	10	9	0.980	16.50	6.00
4	12	3	25	12	3.690	59.00	18.00
5	9	3	10	15	0.980	16.50	6.00
6	9	3	40	9	7.010	110.00	36.00
7	12	2	25	12	0.150	3.00	0.90
8	9	2	25	12	7.010	110.00	36.00
9	12	1	25	12	0.022	2.10	0.12
10	15	3	40	9	0.150	3.00	0.90
11	9	1	10	15	3.690	59.00	18.00
12	15	2	25	12	0.016	1.10	0.10
13	15	1	40	15	5.640	95.30	30.00
14	12	2	25	12	0.150	3.00	0.90
15	9	3	40	15	0.465	8.89	3.00
16	15	3	10	15	0.711	14.50	3.20
17	12	2	25	12	0.024	0.60	0.17
18	12	2	25	9	0.227	4.50	1.50
19	15	3	40	15	0.980	16.50	6.00
20	12	2	25	12	0.150	3.00	0.90
21	9	3	10	9	0.102	1.60	0.60
22	15	1	40	9	0.470	7.50	2.50
23	15	3	10	9	0.330	2.30	1.90
24	12	2	25	12	0.150	3.00	0.90
25	9	1	40	15	4.120	77.00	18.00
26	12	2	25	12	0.150	3.00	0.90
27	15	1	10	15	0.712	10.97	3.50
28	15	1	10	9	2.979	79.67	16.00
29	9	1	40	9	0.980	16.50	6.00
30	12	2	40	12	1.200	16.80	6.20
31	12	2	25	15	0.600	7.90	2.60

#### Key influential parameters and their response surface plots

ANOVA was carried out to identify the significant parameters that influence the performance of MSM under flexural loading, same as done in the previous section for compression loading. ANOVA ensured the accurate prediction of responses as the model was fitted to accurate levels for flexural modulus (FM), and flexural strength (FS) as shown in Table 7.

**Table 7:** Analysis of variance for energy absorbed, modulus and strength under flexural load.

EA	DF	Adj SS	Adj MS	F-Value	*p*-Value
L	1	6.914	6.9142	4.37	0.048
h	1	13.256	13.2561	8.38	0.011
Theta*Theta	1	29.069	29.0693	18.37	0.001
L*Theta	1	13.434	13.4341	8.49	0.010
t*h	1	18.222	18.2222	11.52	0.004
Error	16	25.319	1.5824		
Lack-of-Fit	10	25.319	2.5319	*	*
Pure Error	6	0.000	0.0000		

FM	DF	Adj SS	Adj MS	F-Value	*p*-Value
L	1	2727.6	2727.65	6.83	0.019
t	1	1148.0	1148.00	2.87	0.001
Theta	1	231.1	231.05	0.58	0.458
h	1	4301.3	4301.28	10.76	0.005
Theta*Theta	1	7917.8	7917.84	19.81	0.001
L*Theta	1	4081.6	4081.61	10.21	0.006
t*Theta	1	2367.1	2367.07	5.92	0.027
t*h	1	3710.9	3710.94	9.29	0.008
Error	16	6393.5	399.60		
Lack-of-Fit	10	6393.5	639.35	*	*
Pure Error	6	0.0	0.00		

FS	DF	Adj SS	Adj MS	F-Value	*p*-Value
L	1	160.50	160.50	3.62	0.075
t	1	162.90	162.90	6.90	0.013
h	1	313.75	313.75	7.08	0.017
Theta*Theta	1	748.75	748.75	16.90	0.001
L*Theta	1	370.08	370.08	8.36	0.011
t*h	1	425.49	425.49	9.61	0.007
Error	16	3710.9	231.93		
Lack-of-Fit	10	3710.9	371.09	*	*
Pure Error	6	0.0	0.00		

**Fig. 6:**
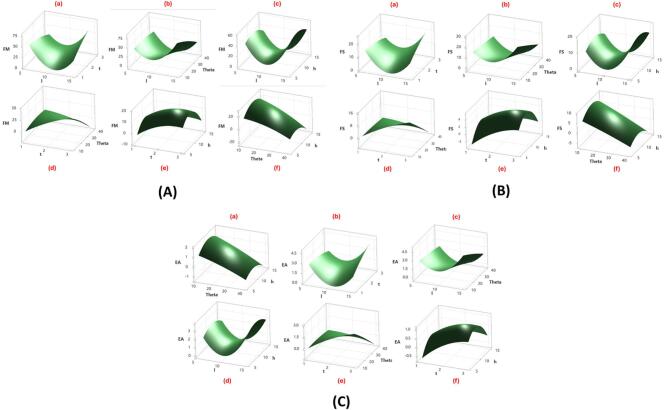
3D surface plots for in bending (**A**) Flexural modulus (**B**) Flexural strength (**C**) Energy absorption.

Table 7 highlights the impact of geometric parameters on MSM for each response, detailing *p*-values and F-values. For EA, length and height emerged as the most significant factors, with *p*-values of 0.048 and 0.011, respectively. In terms of interactions, the combination of theta, length, and thickness was highly significant for square and binary interactions (*p* ≤ 0.05). For flexural modulus (FM), length and thickness were the most significant parameters, with *p*-values ≤ 0.05 and high F-values. Additionally, for FM, the binary and square interactions, along with height alone, were notably significant (*p* = 0.017). These findings confirm the models’ robustness in capturing the trends in the simulation data. Figure 6A–C presents 3D surface plots illustrating the influence of geometric parameters on FM, FS, and EA under bending load. FM increases with thickness and varies nonlinearly with length, while height and angular interactions further contribute to stiffness enhancement. EA is primarily governed by length and height, whereas FS peaks at specific height-angle combinations with intermediate thickness values. Additional surface plots showing similar trends are provided in the supplementary material. Overall, thickness emerged as the most critical parameter, exerting a substantial influence on all measured responses under both compression and flexural loading, followed by length and height, while the inclination angle showed the least impact.

### Performance prediction models of mixed star structure

#### Compression load

The prediction models for the respective responses, based on the CCD for each parameter, are expressed in Eqs. 4–6. These equations predict the mechanical properties for a given set of cell parameters, without conducting physical experiments.

4 $$EA=3.773-0.002l+0.479t+0.330\theta -0.350h-1.505{t}^{2}+0.729l.t-0.975l.\theta -0.895\theta .h$$

5 $$\begin{aligned} CM = & 33.8 + 21.55l + 9.24t + 0.08\theta {\mkern 1mu} \\ & + 7.52h - 19.8t^{2} - 21.9{\mkern 1mu} l{\mathrm{.t}} - 16.8\theta .h + 14.4l.h \\ \end{aligned}$$

6 $$\begin{aligned} CS = & 17.37 + 0.54l + 4.5t - 1.91.h + 14.62l^{2} \\ & - 2.78t^{2} + 9.62h^{2} + 2.63{\mkern 1mu} {\mathrm{L}}{\mathrm{.t}}{\mkern 1mu} - 5.29l.\theta \\ \end{aligned}$$

The ANOVA results, which were shown before in Table 5, provide insights into the second-order regression models for CM, CS, and EA evaluated with a 95% confidence interval. The analysis demonstrates that all the coefficients have statistical significance (*p* ≤ 0.05), each with R^2^ value > 90%, indicating a strong model fit for all responses. Adjusted R^2^ values of 87% for EA and 85%, CM, and 90% for CS further support this fit.

**Fig. 7:**
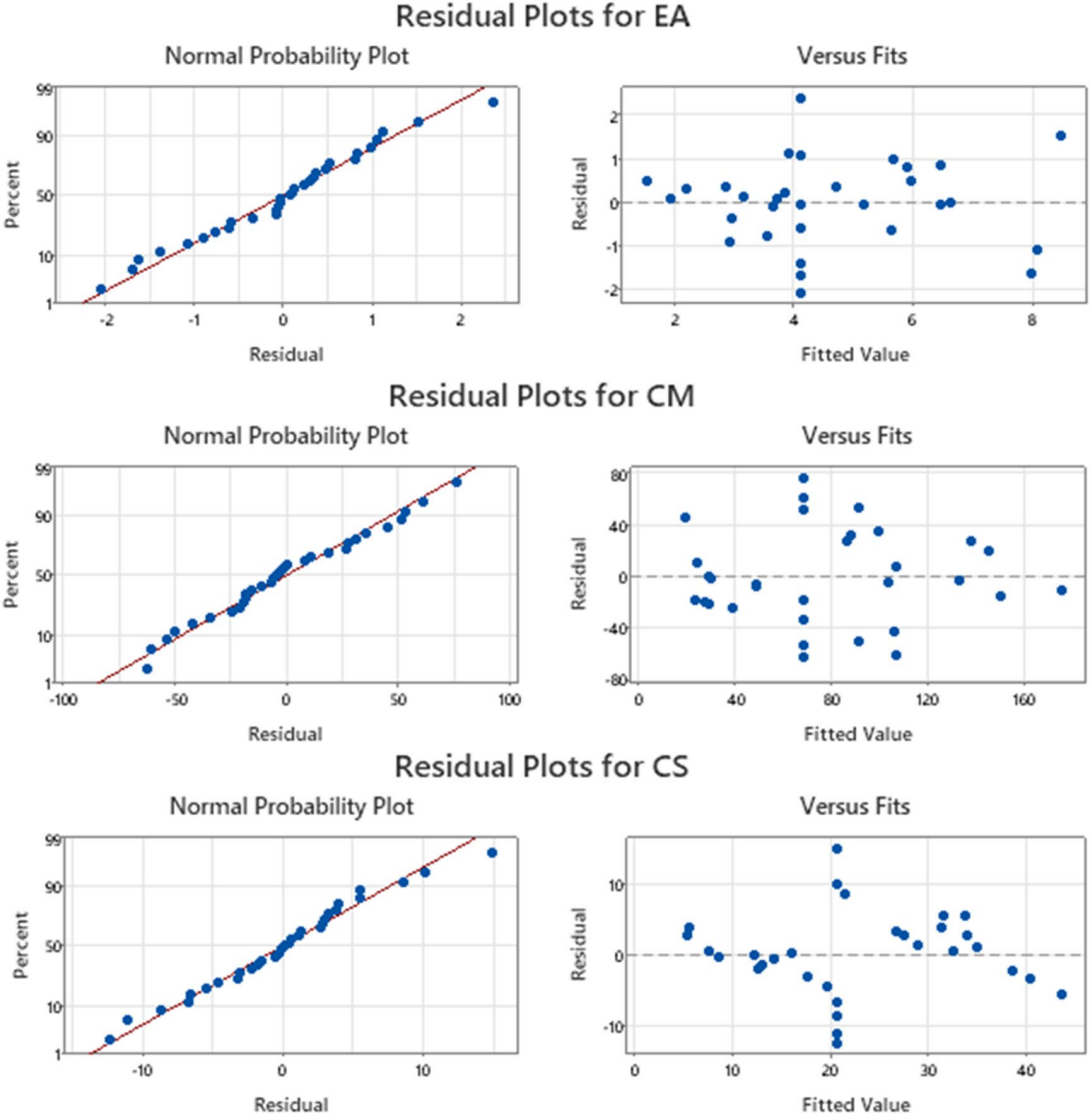
Normality and residual distributions of EA, CM and CS.

The adequacy of regression models was further evaluated through tests for residual normality and homoscedasticity. Figure 7 illustrates that the residuals closely align with the continuous fitted line, indicating that the residuals for each response EA, CM, and CS exhibit acceptable normality. Further validation using the Anderson normality test confirms that the residuals for EA, CM, and CS follow a normal distribution, with *p*-values exceeding 0.05 (0.900 and 0.752, respectively). Additionally, Fig. 7 also reveals that the residuals versus fitted values for EA, CM, and CS are randomly distributed, avoiding cone-shaped patterns. This distribution signifies homoscedasticity, affirming the consistency of residual variance with the fitted values for EA. Therefore, based on these fitness tests, it can be said that the proposed models can be trustfully applied for predicting the compression properties of MSM.

#### Flexural load

The ANOVA results for flexural testing in Table 7 also highlighted the significant parameters with 95% confidence levels for EA, FM, and FS. The models demonstrated strong predictive accuracy, with R^2^ and adjusted R^2^ values of 93% and 90% for EA, 88% and 84% for FM, and 90% and 86% for FS, respectively. The prediction models for the respective responses, based on the CCD for each parameter, are expressed in Eqs. 7–9. These equations predict the mechanical properties for any given parametric values, thereby allowing them to achieve the desired performance.

7 $$EA =1.136+0.620\mathrm{l}-0.590\mathrm{t}-0.131\uptheta +0.858\mathrm{h}+3.347{\uptheta }^{2}-0.916\mathrm{l}.\uptheta -1.067\mathrm{th}$$

8 $$\mathrm{FM}=19+12.1-7.99\mathrm{t}-3.58\theta +15.46\mathrm{h}+55.2{\theta }^{2}-15.971\theta +12.16\mathrm{t}\theta +3.42\theta \mathrm{h}$$

9 $$\mathrm{FS}=6.47+2.991-3.01\mathrm{t}-0.95\theta +4.17\mathrm{h}+16.99{\theta }^{2}-4.811\theta -5.16\mathrm{th}$$

**Fig. 8:**
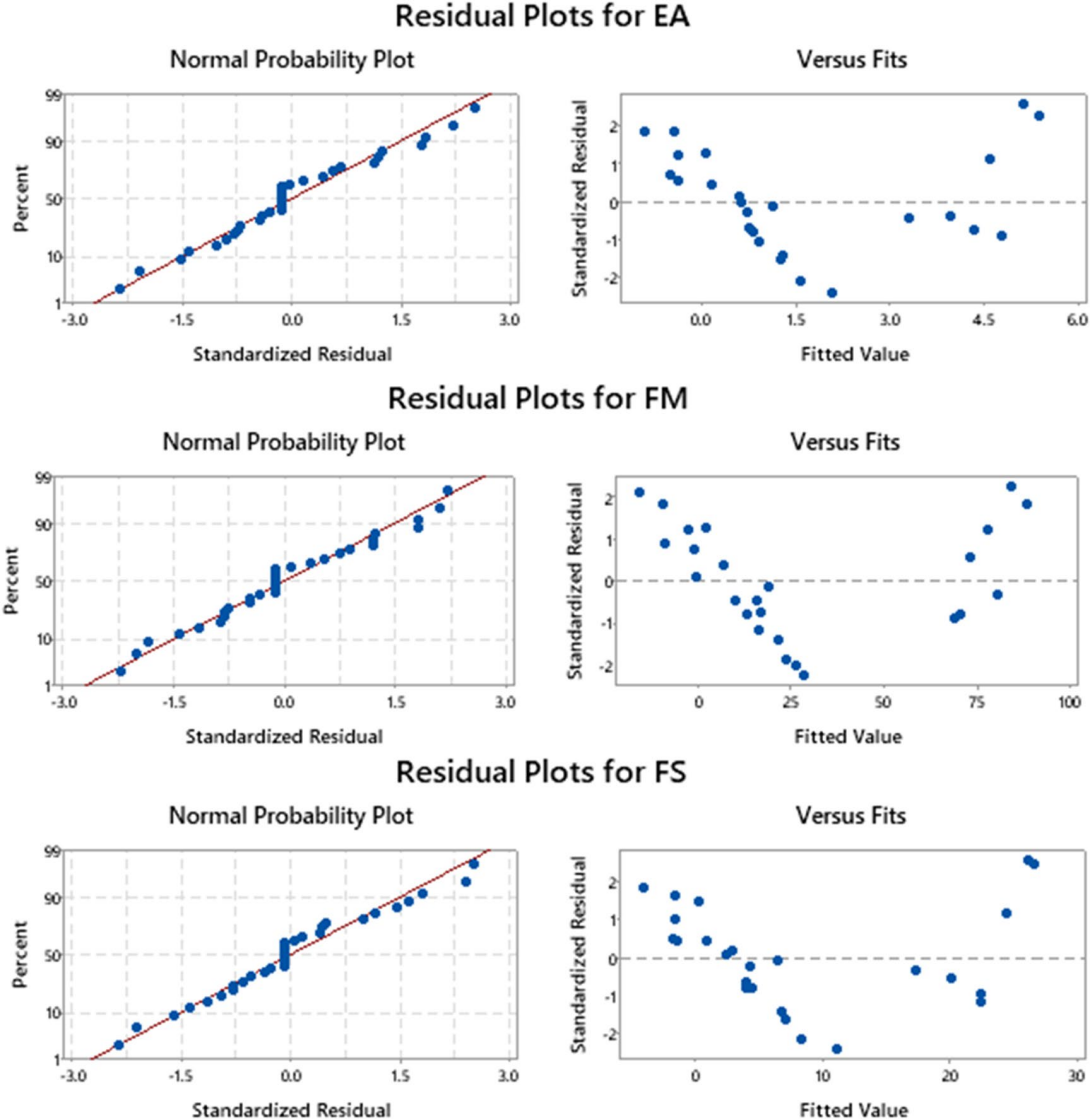
Normal probability and residuals for flexural loading.

The adequacy of the regression models was further assessed through tests for residual normality and homoscedasticity. Figure 8 shows that the residuals closely align with the fitted line, suggesting acceptable normality for each response EA, FM, and FS. The residuals are randomly scattered in the results plot, and the probability distribution follows an inclined line, confirming that all responses adhere to a normal distribution, as insignificant parameters (*p* > 0.05) were excluded. Figure 8 reveals that residuals versus fitted values for EA, FM, and FS are randomly distributed, without cone-shaped patterns. This distribution confirms homoscedasticity, indicating consistent residual variance across fitted values. Based on these fitness tests, it can be concluded that the proposed models are correct and can be reliably used for predicting the flexural properties of MSM.

### Cell optimization

**Fig. 9:**
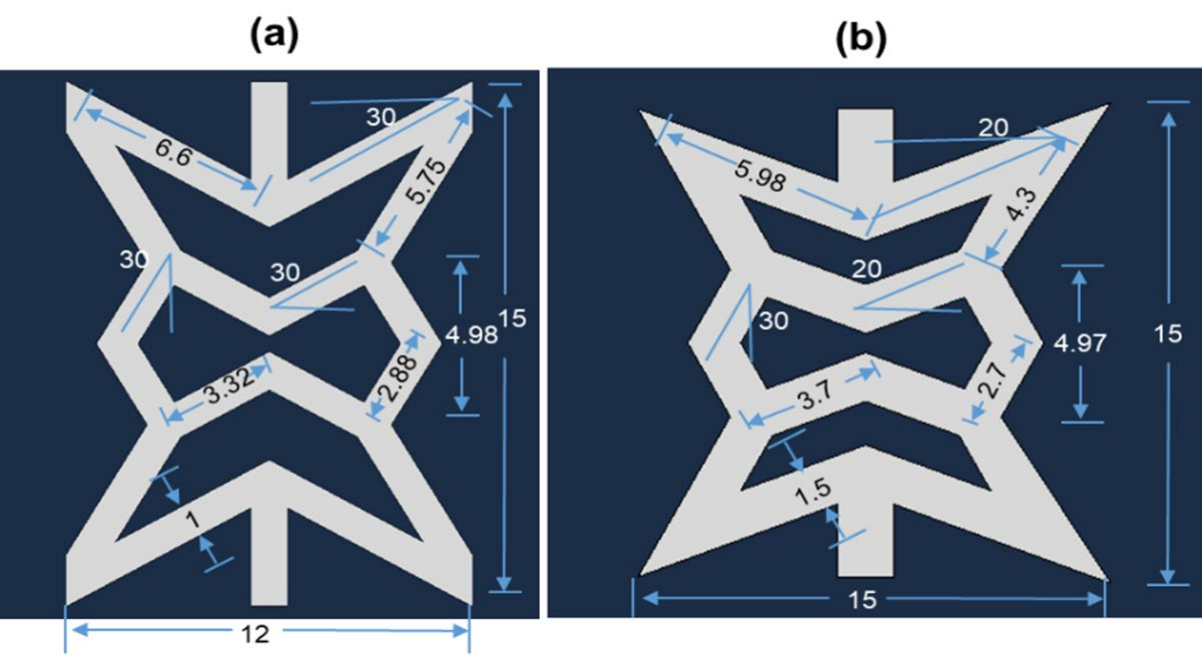
MSM according to optimized parameter.

The shape of the final geometry modeled according to the optimized cell geometry is shown in Fig. 9. Table 8 lists the geometrical parameters of optimized and unoptimized cells. As the optimum cell parameters are comparable for both types of loads, this suggests that the optimized structure can perform well under combined compression-bending load. The optimized cell geometry for both compression and bending loading conditions converged to identical parameter values. This convergence can be attributed to the dominant influence of thickness, length, and height on the mechanical response of the MSM structure, as these parameters govern ligament stiffness, load distribution, and buckling resistance under both loading modes. Consequently, a single optimized geometry yields balanced improvements in stiffness, strength, and energy absorption regardless of the loading condition. The consistency of optimal parameters across both loading conditions highlights the robustness of the proposed optimization framework.

**Table 8:** Cell parameters of optimized MSM.

	Relative density	$h$(mm)	${h}_{1}$(mm)	${l}_{1}$(mm)	${l}_{2}$(mm)	${l}_{3}$(mm)	${l}_{4}$(mm)	$t$(mm)	${\theta }_{1}$	${\theta }_{2}$	${\theta }_{3}$
Compression											
Un-optimized	0.43	15	4.98	5.75	6.6	2.88	3.32	1	30º	30º	30º
Geometrically optimized	0.75	15	4.97	5.98	4.3	2.71	3.7	1.5	20º	20º	30º
Bending											
Un-optimized	0.43	15	4.98	5.75	6.6	2.88	3.32	1	30º	30º	30º
Geometrically optimized	0.75	15	4.97	5.98	4.3	2.71	3.7	1.5	20º	20º	30º

**Fig. 10:**
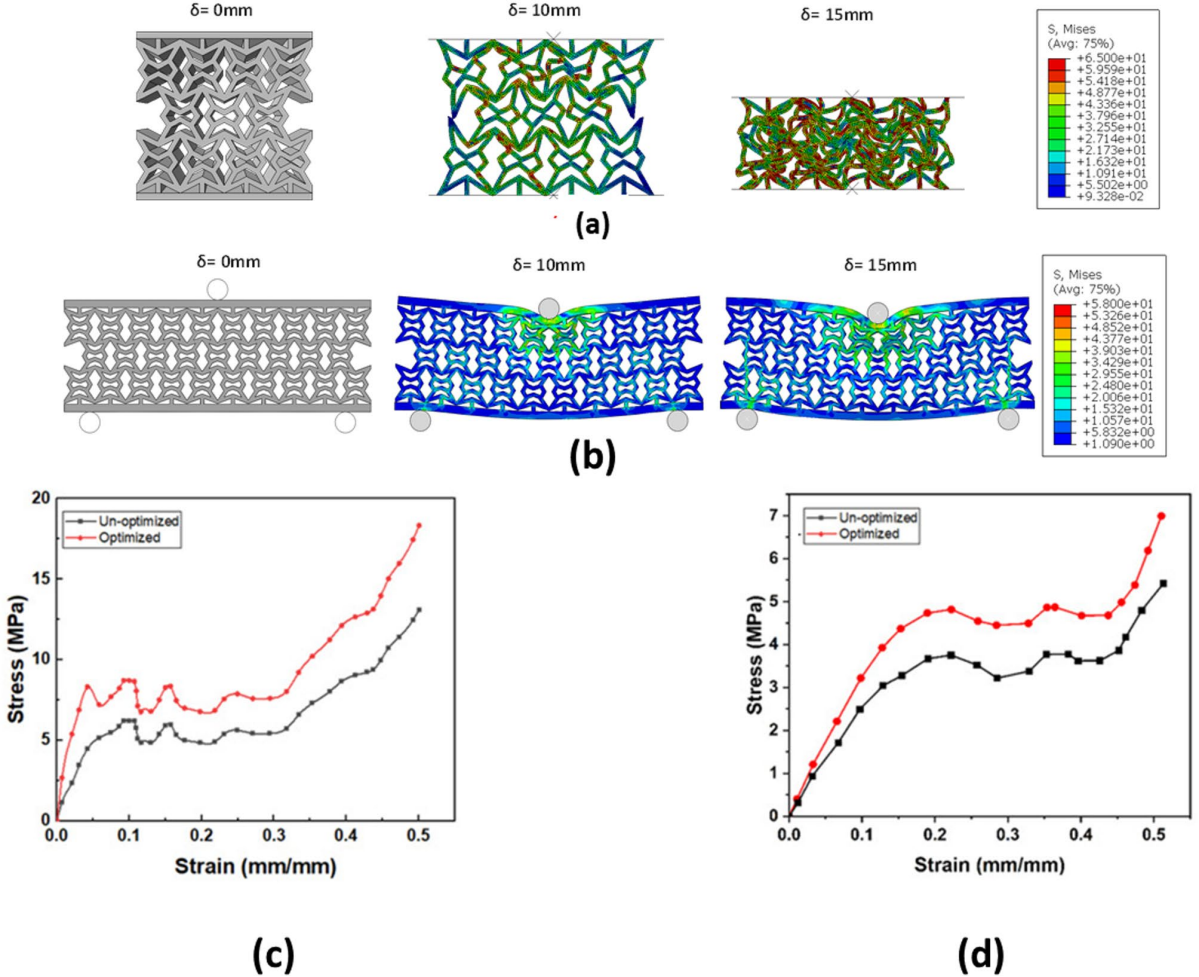
Optimized structure: (**a**) Under compression (**b**) Under bending (**c**) Stress–strain curve for unoptimized and optimized MSM for compression (**d**) for flexural.

The mechanical properties of optimized structures, predicted via a statistical model, are shown in Table 9. However, to ensure that these predicted values are correct, actual testing was performed using the FEA methodology. The structures for compression and bending were modeled using optimized cell parameters, and the FEA simulations were performed following the procedure detailed in "Predictive models" section. The corresponding stress–strain curves were derived from the simulation results shown in Fig. 10a-b. In Fig. 10a stress was initially concentrated in the top row of the structure under compression load. As it progressed toward the middle row, the cell walls began to buckle. At a displacement of 5 mm, the cell walls started to collapse, but as the displacement reached 15 mm, the structure underwent densification, resulting in an increased load-bearing capacity. Under flexural loading, Fig. 10b illustrates the deformation behavior of the MSM under three-point bending. Initial deformation occurs in the first layer, which collapses at a displacement of 5 mm. As stress propagates, subsequent cell walls also collapse. The optimized structure demonstrates enhanced load-bearing capacity, attributed to the increased thickness of the cell walls, which improves stiffness and effectively resists stress propagation.

**Table 9:** Predicted and actual results of optimized geometry.

Mechanical properties	Predicted results	FEA results	% Error
Compression			
EA (× 10^3^ J/m^3^)	24.1	25	4.1
CM (MPa)	181.5	180	0.83
CS (MPa)	8.9	8.4	5
Flexural			
EA (× 10^3^ J/m^3^)	7.23	7.52	3.86
FM (MPa)	10.4	9.89	4.9
FS (MPa)	4.1	4.31	5

Table 9 compares the predicted and actual results. As is clear, the error between the predicted and actual values of the considered mechanical properties is 5% (max). Therefore, it can be claimed that the proposed optimum geometry is valid for real applications.

**Fig. 11:**
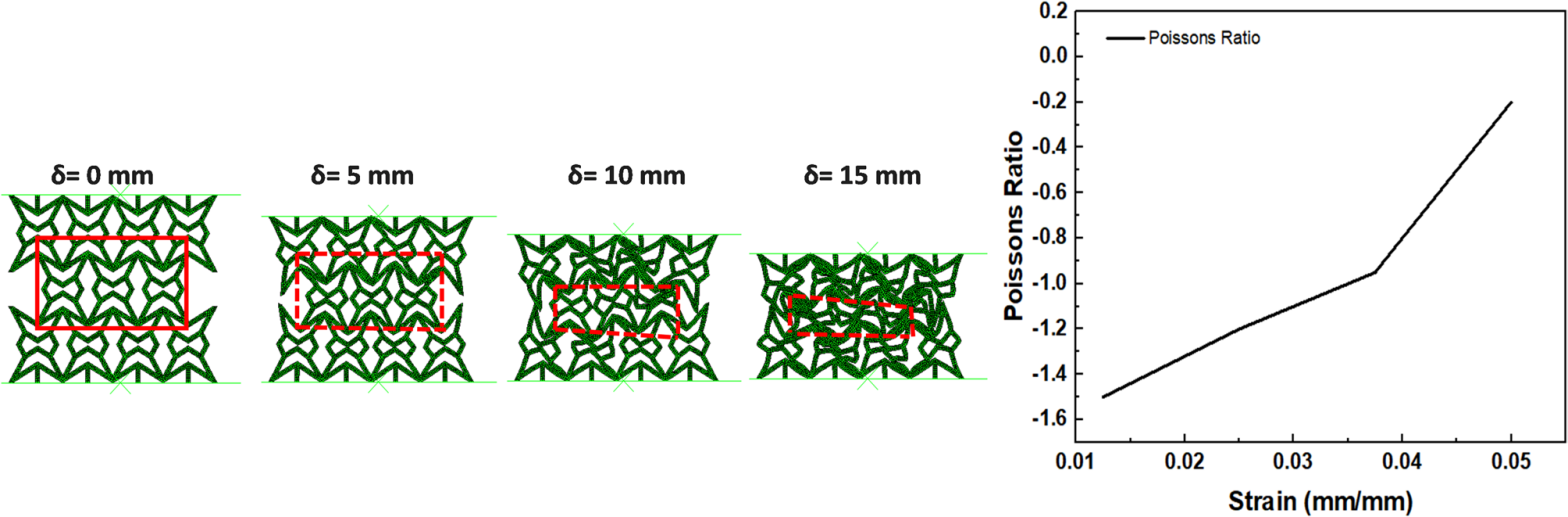
Auxeticity of MSM at different strain.

Poisson’s ratio for both compression and bending of MSM was determined using the methodology described in^52,56^. A rectangular block was positioned at the center of the structure, and its initial and final dimensions were measured using Image J to evaluate auxetic behavior, confirming a reduction in lateral dimensions under compression and bending. The deformation analysis demonstrated that the MSM geometry exhibited auxetic behavior under both loading scenarios. Figure 11 illustrates that the auxeticity is maximum to -1.5 when the load is initially applied leading to decrease in auxeticity towards the densification phase but still possess NPR up to -0.2. When compared to unoptimized geometry, the optimized structure showed a significant improvement in auxeticity. Under compression, the auxeticity of the optimized MSM reflects a 25% enhancement as compared to unoptimized MSM. Similarly, under bending, the optimized MSM achieved an auxeticity of a 31% improvement as compared to unoptimized MSM. These results clearly demonstrate that geometry-optimized MSM outperformed the unoptimized design in terms of auxetic behavior.

### Performance gains of geometrically optimized MSM

The stress–strain curve for compression in Fig. 10a demonstrates that the optimized structure exhibits significantly improved mechanical performance compared to the unoptimized one. The linear region indicates elastic deformation, where the structure resists compressive loads without permanent deformation. As strain increases, the optimized structure maintains a higher stress-bearing capacity, reflecting enhanced stiffness and resistance to collapse. Figure 10b shows the stress–strain behavior under flexural loading. The linear region indicates high flexural strength, contributing to a high flexural modulus. As strain progresses to 0.35, a slight hump appears due to cell deformation. When cells come into contact, the strength increases, resulting in continuous stress-bearing behavior until each cell deforms and densification begins, enhancing the structure’s load-bearing capacity as densification progresses. This improved behavior can be attributed to structural modifications, such as increased wall thickness or cell optimization, which contribute to better load distribution and resistance to densification.

A comparison of optimized and unoptimized MSM is presented in Table 10. The results show that the geometrically optimized MSM has performed better than the un-optimized structure reported in our previous work^50,57^ based on MSM in all desired mechanical responses. The results show an increase in EA of 624% followed by the CM and CS with an increase in performance with a significant 312% to 115% respectively for the compression performance. As for the flexural performance, the trend was the same as the EA was seen to increase by 743% and FM and FS increased by 160% and 238% respectively. The observed improvements can be attributed to geometric optimized MSM enhanced resistance to deformation, resulting in stiffer and more robust load-bearing structures.

**Table 10:** Comparative analysis of unoptimized and optimized MSM.

Mechanical properties	Unoptimized	Geometric optimized	% Increase
Compression			
EA (× 10^3^ J/m^3^)	3.93	24.1	624
CM (MPa)	43.9	181.5	312
CS (MPa)	4.23	9.1	115
Flexural			
EA (× 10^3^ J/m^3^)	0.86	7.23	735
FM (MPa)	8.51	20.8	160
FS (MPa)	1.21	4.1	238

Table 11 shows the comparative analysis of the optimized MSM structure with other auxetic geometries under compression loading made of ABS material. Poisson’s ratio of MSM is the most negative one (− 1.2), which shows better auxetic properties than others. The modulus of MSM (181.5 MPa) is also higher than the re-entrant (32 MPa), chiral (4 MPa), and tetra-chiral (28.9 MPa) geometries. Although MSM has higher modulus values, its EA (24 J/m^3^) is higher than the re-entrant and tetra-chiral geometries. This comparison proves the better mechanical and energy absorption properties of the MSM structure.

**Table 11:** Comparison of Poisson’s ratio, modulus, EA for MSM and auxetic geometries.

Structure	Poisson’s Ratio (ν)	Modulus (MPa)	EA (J/m^3^)
MSM	− 1.5	181.5	24
Re-entrant^50^	− 0.3	32	0.37
Chiral^20^	− 0.4	4	9.8
Honeycomb^58^	0.3	3.5	6
Tetra chiral^56^	− 0.6	28.9	2.7

## Conclusions

Auxetic structures owing to negative Poisson’s ratio can satisfy the increasing demand for lightweighting with high mechanical performance. To further enhance their structural integrity, innovative designs and approaches should be proposed. The present study, therefore, introduces and optimizes the design of such a structure named Mixed Star Structure (MSM). Its cell geometry is optimized, adopting a hybrid statistical-numerical approach, to maximize the mechanical performance under compression and flexural loads. The structural performance was measured in terms of energy absorbed, modulus, and strength. The following important findings can be derived from the study:

- Cell optimization and auxeticity (negative poison’s ratio) in structures were found to be closely related. Compared with unoptimized ones, the auxeticity of optimized cells increased by 25% and 31% in compression and bending, respectively. As a result, the MSM exhibited substantial performance gains, namely 624% in energy absorption in compression, 312% in compression modulus, and 115% in compressive strength. The corresponding gains recorded for flexural loading are 743%, 160%, and 238%.
- The cell wall thickness ($t$), unit cell length ($L$), and cell height ($h$) showed the most significant impact on mechanical performance. As a result of varying these parameters, performance ranged in a wide range. For compression, energy absorbed ranged from 2.01 to 24.1 kJ/m^3^, modulus ranged from 5.55 to 181.5 MPa, and strength ranged from 0.3 to 9.1 MPa. Similarly, for bending load, energy absorbed ranged from 0 to 7.23 kJ/m^3^, modulus ranged from 0 to 20.8 MPa, and strength ranged from 0 to 4.1 MPa. These variations in properties are mainly due to change in auxeticity.
- The four cell parameters investigated, namely length, height, inclination angle and wall thickness, affected the structure’s performance. However, the wall thickness appeared as the most important one in both the compression and flexural loadings. Moreover, each one was found to be interacting with the other cell parameters in regard to its effect. The response to a variation in the cell size was found to be complicated and dependent on the type of loading thereby calling for geometry optimization to realize load-specific optimum performance.
- This study applied a cost-effective hybrid statistical-numerical approach to evaluate the structure’s performance and to optimize the cell geometry. The optimize cell size for MSM is as follows: t = 1.5 mm, h = 15 mm, θ = 20°, and l = 5.98 mm. Interestingly, the same constituted an optimum cell size for flexural loading. This follows that the proposed optimized MSM structure is capable of performing equally well under both types of loads. However, further work considering a variety of structures and loads is required to generalize this finding.
- Prediction models are proposed here in study. These models, as confirmed through several tests, are fairly accurate in predicting the properties thereby confirming that the hybrid statistical-numerical approach is an effective tool for geometrically optimizing and advancing auxetic structures for structural applications.

## Data Availability

Data will be made available upon request.
